# Distinct Patterns of Brain Activity Characterise Lexical Activation and Competition in Spoken Word Production

**DOI:** 10.1371/journal.pone.0088674

**Published:** 2014-02-18

**Authors:** Vitória Piai, Ardi Roelofs, Ole Jensen, Jan-Mathijs Schoffelen, Mathilde Bonnefond

**Affiliations:** 1 Donders Institute for Brain, Cognition and Behaviour, Radboud University Nijmegen, Nijmegen, the Netherlands; 2 International Max Planck Research School for Language Sciences, Max Planck Institute for Psycholinguistics, Nijmegen, the Netherlands; 3 Neurobiology of Language Department, Max Planck Institute for Psycholinguistics, Nijmegen, the Netherlands; Utrecht University, Netherlands

## Abstract

According to a prominent theory of language production, concepts activate multiple associated words in memory, which enter into competition for selection. However, only a few electrophysiological studies have identified brain responses reflecting competition. Here, we report a magnetoencephalography study in which the activation of competing words was manipulated by presenting pictures (e.g., dog) with distractor words. The distractor and picture name were semantically related (*cat*), unrelated (*pin*), or identical (*dog*). Related distractors are stronger competitors to the picture name because they receive additional activation from the picture relative to other distractors. Picture naming times were longer with related than unrelated and identical distractors. Phase-locked and non-phase-locked activity were distinct but temporally related. Phase-locked activity in left temporal cortex, peaking at 400 ms, was larger on unrelated than related and identical trials, suggesting differential activation of alternative words by the picture-word stimuli. Non-phase-locked activity between roughly 350–650 ms (4–10 Hz) in left superior frontal gyrus was larger on related than unrelated and identical trials, suggesting differential resolution of the competition among the alternatives, as reflected in the naming times. These findings characterise distinct patterns of activity associated with lexical activation and competition, supporting the theory that words are selected by competition.

## Introduction

A core process in spoken language production is the quick and accurate retrieval of intended words from long-term memory. According to a prominent theory [1]–[5], conceptually driven word retrieval involves the activation of a set of candidate words in left middle temporal cortex, and competitive selection of the intended word from this set regulated by frontal cortical mechanisms. However, although competition is widely regarded in the cognitive neurosciences as a ubiquitous mechanism [6], [7], its role in lexical selection has recently been disputed [8]–[11]. Whereas electrophysiological studies have provided evidence for the activation of multiple lexical candidates, no study so far has explicitly identified brain responses reflecting the top-down (i.e., from frontal brain areas) resolution of lexical competition. Here, we provide evidence from magnetoencephalography (MEG) that evoked (i.e., phase-locked) activity in left temporal cortex and induced (i.e., non-phase-locked) activity in superior frontal cortex characterise, respectively, lexical activation and competition in overt picture naming, thereby supporting the theory of lexical selection by competition.

Earlier behavioural evidence for multiple lexical activation and competition comes from studies of picture naming in which the amount of lexical competition is manipulated by simultaneously presenting distractor words. These words may be semantically related (e.g., a picture of a dog combined with the word *cat*), unrelated (pictured dog, word *pin*), or identical (pictured dog, word *dog*) to the picture name. Picture naming response time (RT) is typically longer in the related than in the unrelated condition, referred to as the *semantic* effect, and longer in the related than in the identity condition, referred to as the *Stroop-like* effect [12], [13]. According to the theory [1]–[5], a picture (e.g., of a dog) activates, to different degrees, multiple lexical candidates that are semantically related (e.g., *dog*, *cat*, *goat*, etc.). In particular, the picture (e.g., of a *dog*) will prime the distractor word (e.g., *cat*) via conceptual connections in memory, referred to as *reverse priming* [14], [15], and the distractor word will prime the picture name. Consequently, a semantically related distractor word (e.g., *cat*) receives further activation from the picture (dog) and is therefore a stronger competitor to the picture name than an unrelated distractor word (e.g., *pin*), which is not activated by the picture. When picture name and distractor are identical (*dog*), activation of the intended word will be increased relative to alternative words. The enhanced activation of the distractor word in the related condition compared with the other conditions prolongs the duration of word selection and yields the semantic and Stroop-like interference effects in the RTs. Thus, the semantic (related vs. unrelated) and Stroop-like (related vs. identity) effects reflect the involvement of competition in lexical selection [16], [17]. The account of lexical selection in terms of activation (reverse priming) and competition has been implemented in computational models of word production, including the model of Starreveld and La Heij [18], and WEAVER++ (e.g. [2]–[4], [16], [19], [20]), which successfully simulate a wide range of findings in the literature on spoken word production (e.g. [2], [3], [16]).

Previous electrophysiological (EEG) studies examining lexical selection in picture naming have provided evidence for the activation of multiple lexical candidates [8], [21]. These studies observed an N400 response, which is a broad negative-going event-related potential (ERP) that usually peaks at approximately 400 ms post-stimulus onset [22]–[24]. Generally, the amplitude of the N400 response seems to reflect the ease of integration of or access to stored representations [23], [24]. In particular, semantically primed stimuli elicit an attenuated N400 response relative to unprimed stimuli (for review ref. [23]). In picture naming with distractor words, the amplitude of the N400 tends to be larger in the unrelated than in the related and identity conditions, i.e., unrelated > related > identity ([8], [21], [25], [26] but note that ref. [8] did not use a conventional picture-word interference paradigm), suggesting the activation of multiple lexical alternatives. The co-activation of semantic alternatives (due to priming) reduces the effort of processing the picture name (*dog*) and the distractor word (*cat*) in the related condition relative to the unrelated condition (*pin*), where there will be no such co-activation. When picture name and distractor word are identical, their activation converges on a single word in memory (*dog*), reducing processing effort even further.

However, activation of multiple lexical candidates does not necessarily imply that the selection of the intended word is a competitive process [8], [11]. On an alternative account, picture and word also prime each other in the related condition [10]. However, candidate words do not enter into competition but rather the first word that exceeds an activation threshold is selected [9], [10], [27]. Under this account, the semantic and Stroop-like effects arise when an articulatory programme derived for the distractor word needs to be excluded from an articulatory buffer to give place to the articulatory programme for the picture name (e.g. [9], [28]). The decision mechanism that excludes the programme for the distractor from the buffer is assumed to be sensitive to whether the distractor word belongs to the same semantic category as the picture, explaining the semantic and Stroop-like effects in the RTs.

The ERP findings in the literature may have provided evidence for the co-activation of lexical candidates, but only a few studies have identified increased brain responses that are analogous to the increase in RTs for the related condition compared with the unrelated and identity conditions [29]–[31]. According to Blackford et al. [8], the finding of an attenuated N400 (related < unrelated ERP amplitudes) associated with increased RTs in the related condition (related > unrelated RTs), as observed in the literature [8], [21], challenges the theory that competition is involved in lexical selection [1]–[5]. However, the conclusion of Blackford et al. seems to be challenged by other evidence in the literature indicating positive correlations between semantic interference effects in the RTs and EEG modulations (i.e., related > unrelated; e.g., in continuous/cyclic semantic blocking paradigms and picture-word interference tasks; (e.g. [29], [30])).

Importantly, ERPs are calculated by averaging, over several trials, the EEG signal time-locked to a stimulus. This may capture electrophysiological activity that is phase-locked to the stimulus, referred to as *evoked activity*, but will miss brain activity that is not phase-locked to the stimulus, referred to as *induced activity* [32]. Induced activity may be examined, though, by means of time-frequency representations (TFRs), which capture changes in oscillatory brain activity over time, regardless of phase locking. Previous research suggests that evoked and induced activity may reflect largely distinct functional processes [32], [33]. In particular, whereas bottom-up processes, like memory activation in the present context, can be reflected in evoked and induced activity, induced activity seems to be more dependent on top-down processes [32], [34], like executive control over memory representations in the present context. Resolving lexical competition requires top-down executive control over activated lexical candidates [3]–[5], [35], [36]. In short, previous EEG studies that reported an attenuated N400 amplitude associated with the semantic interference effect in RTs (e.g. [8], [21]) may have failed to find evidence for competition because they examined evoked brain activity only.

The present study aimed at an electrophysiological characterisation, both in time and in terms of involved brain areas, of the competition that is triggered by the semantic co-activation of lexical candidates. Participants overtly named pictures, while trying to ignore distractor words that were semantically related (e.g., a picture of a dog combined with the word *cat*), unrelated (*pin*), or identical (*dog*). We used MEG to examine evoked and induced activity associated with distractor effects. Changes in event-related fields (ERFs, the magnetoencephalographic equivalent of ERPs) were expected to reflect the activation of multiple candidates [8]. The neuronal generators of the N400 effect in picture-word interference studies are unknown. However, the activation of multiple lexical candidates in picture naming has been associated with left middle temporal gyrus (MTG) [1], [2], [37], [38]. Based on earlier ERP studies, we expected the ERF amplitude in left MTG to be larger in the unrelated than in the related and identity conditions [8], [21], [25], [26]. The induced activity, in turn, was expected to reflect competition resolution processes. Although very little is known about oscillations in picture naming [26], [33], [39], power modulations in the theta (4–7 Hz) and alpha (8–12 Hz) frequency bands have been observed in a color-word Stroop analog of picture-word interference using manual responding [40]. Competition effects in Stroop-like tasks are typically localised to frontal cortex [41], which is also associated with executive control in word production [3]–[5]. Therefore, we expected competition resolution in picture naming to be reflected in induced activity in a frequency band between 4–12 Hz in frontal brain areas. Activity should be larger for the related than unrelated and identity conditions, corresponding to the condition ordering of the mean RTs.

According to the noncompetitive account of word retrieval [9], [10], [27], [28], the interference in the naming RTs arises *after* word planning, in an articulatory buffer, “at the point of deciding which of two articulatory programs should be excluded from the output buffer in order that the correct response may be produced” (ref. [10], p. 1033). Importantly, meta-analyses have provided time estimates indicating that an articulatory programme reaches the buffer no earlier than about 145 ms before articulation onset [37], [38]. We used response-locked analyses to assess whether modulations of induced brain activity happen later than 145 ms before articulation onset, as predicted by the noncompetitive account [9], [10], [27], [28], or earlier in time, as predicted by the lexical competition account [1]–[5]. Response-locked analyses have been proposed as a tool to help adjudicate between the two accounts: “Additional methods of analysis, examining […] backwards from naming onset, will be required to determine whether […] behavioral semantic interference occur at intermediate stages or at very late stages of processing during preparation of the articulatory response.” (ref. [8], p. 97).

## Methods

### Ethics Statement

This study was approved by the Ethics Committee for Behavioural Research of the Social Sciences Faculty at Radboud University Nijmegen and followed the Declaration of Helsinki (World Medical Association 1964, 2008).

### Participants

Seventeen healthy right-handed, Dutch adults (6 male, mean age  = 21.8, *sd* = 3.5) voluntarily participated in the experiment for monetary compensation or for course credits. All participants had normal or corrected-to-normal vision, and no history of neurological or language deficits. Participants gave written consent after they were completely informed about the nature of the study.

### Materials, Design and Behavioural Procedure

Thirty-six line drawings of common objects, belonging to nine different semantic categories, were taken from the picture database of the Max Planck Institute for Psycholinguistics, Nijmegen. The materials are listed in Table S1. Each picture was paired with a distractor word. In the identity condition, the distractor was the picture's Dutch basic-level name. For the related condition, picture names from the same semantic category were used, and from a different category in the unrelated condition. Thus, our distractor words were part of the response set. All picture-word pairs were presented four times each. Thus, all participants saw all pictures in all conditions, with one unique randomization per participant. Participants were instructed to name the pictures and to ignore the words. Next, they were familiarised with the pictures and their names. After a short practice with 10 trials, the experiment proper started. A trial began with a fixation cross centred on the screen for 1.75 s, followed by the stimulus for 1.5 s. Three asterisks followed, indicating a blinking moment for 1.5 s, followed by an empty screen for 0.5 s. The trials were divided into eight blocks with self-paced breaks in between.

### MEG Procedure

The MEG system (CTF VSM MedTech) contained 275 axial gradiometers. The horizontal and vertical electrooculogram was recorded using two pairs of Ag/AgCl-electrodes. Surface electromyogram was recorded from the orbicularis oris muscle (electrode placement: left upper and right lower corner of the mouth). Three localisation coils were fixed to the nasion, left, and right ear canal to monitor the position of participants' heads relative to the gradiometers. Head localisation was performed in real-time and the head position was re-adjusted when needed to remain in the initial position [42]. The data were low-pass filtered by an anti-aliasing filter (300 Hz cutoff), digitised at 1200 Hz, and stored for offline analysis. A microphone in the magnetically shielded room was connected to a computer, which controlled stimulus presentation with the software package Presentation (Neurobehavioral Systems). Anatomical MRIs of the participants' brains were acquired with a 1.5 T Siemens Magnetom Sonata system. To optimise the alignment of the MRI with the MEG data, the same ear plugs were used during the MEG session and the MR session.

### RT Analysis

Vocal responses were evaluated in real time. Responses containing disfluencies or errors were coded as invalid, analysed separately with logistic regression for accuracy, and their corresponding trials excluded from all subsequent analyses. We submitted RTs to analyses of variance on the average naming RTs across participants (*F*_1_) and across items (*F*_2_), with distractor type as an independent variable. Paired-samples *t*-tests were used to evaluate the Stroop-like (related vs. identity) and the semantic (related vs. unrelated) effects with Bonferroni correction for two comparisons. Additionally, 95% confidence intervals around the mean, calculated from the variance over participants, are reported.

### MEG Data Analysis

#### Preprocessing

The MEG analyses were performed using FieldTrip [43]. The data were down-sampled offline to 600 Hz. Power line fluctuations were estimated and subtracted from the data by fitting narrow-band sinusoidal functions at 50, 100 and 150 Hz. For the stimulus-locked analyses, the data were segmented into epochs from 1 s pre-stimulus to 1 s post-stimulus. For the response-locked analyses, we segmented the data by using the RT of each individual trial. The resulting epochs ranged from 1 s before the response until the RT itself, now the 0-ms point. All epochs were inspected individually. Epochs containing ocular artefacts, SQUID jumps, and mouth EMG artefacts were detected based on sudden deviations from the ongoing signal and localisation on sensors, and subsequently removed (27% of the data, including trials excluded from the RT analysis). Excessively noisy channels were also removed.

#### Sensor-level analysis

Synthetic planar gradients were calculated [44], on which all subsequent sensor-level analyses were performed. Using the combined planar gradient representation of the magnetic fields, the amplitude of the signal on the scalp is largest above the actual sources, facilitating the interpretation of sensor topographies. Moreover, sensor-level group analysis is facilitated and statistical sensitivity is increased.

##### Induced activity

Only the stimulus time-locked trials with RTs larger than 600 ms were entered in the analyses to prevent contamination of the signal with motor artefacts. For the stimulus-locked activity, TFRs of power were computed between 200 ms pre- to 1 s post-stimulus, at frequencies between 2 and 30 Hz. For the response-locked analysis, TFRs of power were computed over the whole segment length, at frequencies between 2 and 30 Hz. We used a sliding time window of three cycles' length (e.g., the window was 300 ms long at 10 Hz), advancing in steps of 50 ms and of 1 Hz. (The value of 1000 ms post-stimulus was chosen to allow for an estimation of three cycles of theta activity until 800 ms.) The data in each time window was multiplied with a Hanning taper before estimating power with the fast Fourier transform (FFT).

##### Evoked activity

Only the stimulus time-locked trials with RTs larger than 600 ms were entered in the analyses to prevent contamination of the signal with motor artefacts. The same number of trials for each distractor type was used (excessive trials were excluded randomly). Epochs were segmented consisting of 200 ms pre- to 800 ms post-stimulus (chosen for being shorter than the mean RTs). The data were filtered with a low-pass filter of 20 Hz and baseline corrected with the 200 ms pre-stimulus interval.

##### Statistical analysis

The sensor-level effects were statistically tested using a non-parametric cluster-based permutation approach [45]. This test provides a significant cluster (corrected for multiple comparisons) of adjacent time-points, sensors (and frequencies) that exhibit a similar difference across conditions. Given the hypothesis that the evoked activity in picture-word interference is similar to the classical N400, we constrained the analyses of the ERFs to a time window (350–550 ms) associated with the N400 effect [23], [24], and to all left temporal MEG sensors [24] that were available for all participants, following demonstrations that the N400m is especially prominent over left-temporal sensors [46], [47]. For the TFRs, given the lack of a-priori hypotheses, whole time epochs and all sensors that were available for all participants were entered in the analyses, but the frequency range was constrained to 4–12 Hz [39], [40].

#### Source-level analysis

The source-level analyses were conducted in the following way.

##### Anatomical processing

Due to technical failures during the measurements, head localisation was not performed for three participants, so the source-level analyses comprised 14 participants. From each participant's anatomical MRI, after segmentation using SPM, we constructed a realistically shaped single-shell model of the inside of the skull, serving as the volume conduction model. This triangulated boundary was subsequently used in combination with a geometric description of the potential neuronal sources (the source model) to compute the forward model [48]. For the reconstruction of the evoked activity we estimated the minimum-norm solution of a distributed source model, based on the individual cortical sheet, reconstructed using Freesurfer [49] and downsampled to 8196 dipole locations using MNE-suite (Hämäläinen, Martinos Center for Biomedical Imaging, Massachusetts General Hospital, MA). For the reconstruction of the induced activity we used beamformers, scanning through a regular 3-dimensional grid of source locations with 1 cm resolution. Beamformers are especially suitable for analysing oscillatory activity [50], but less so for evoked responses. Thus, we used the most suitable type of method for each type of activity (for a similar approach ref. [33]).

##### Induced activity

Source-level theta-band power was estimated using frequency domain beamforming [51]. A multitaper FFT with 2 Hz smoothing was applied to each trial segment (354–640 ms), and we selected the frequency bin centred at 7 Hz. The time window was chosen for being suitable for 2 cycles of 7 Hz oscillations. From the Fourier representation, the sensor-level cross-spectral density matrix was computed (for each effect we combined the two contrasted conditions in order to estimate the spatial filters specific for each effect), and the cross-spectral density matrices were used in combination with the leadfields to compute the spatial filters at each location of the 3-dimensional grid. The spatial filters were then applied to the Fourier transformed data from the individual conditions, allowing for a power estimate for each grid point, per participant, and per condition. The source locations showing local maxima over the whole brain in the reconstructed theta power were selected for further analysis (sources of interest). Using linearly constrained minimum variance beamforming [52], we estimated the time course of the activations of neural sources at the selected locations. TFRs of the reconstructed activity were obtained using the same parameters as for the sensor-level TFRs. We used the time-frequency window of the significant theta activity on the sensor level (350–650 ms) to compute an average for each estimated source per participant. The averaged activity was tested with one-tailed paired-samples *t*-test for the Stroop-like (related > identity) and the semantic (related > unrelated) effects.

##### Evoked activity

The same trials entered in the sensor-level analyses were used for the minimum-norm reconstruction, but the epochs were further constrained from 200 ms pre- to 600 ms post-stimulus to avoid contamination from speech artefacts. The noise-covariance matrix was estimated based on the data from whole epochs (−200 to 600 ms) across distractor-type conditions and was used to regularise the inverse solution, and to compute noise-normalised estimates of neural activity. For the subsequent group analysis, the resulting estimates of neural activity were interpolated onto a regular 3-dimensional grid (8 mm resolution) and normalised to the MNI template brain, using SPM. First, a whole-brain analysis was conducted to identify brain areas associated with the modulations of the evoked activity as a function of distractor type. Based on the time windows identified as significant in the sensor-level analyses for each effect separately, the interpolated and normalised minimum-norm estimates were averaged for each condition separately. The averaged activity was then contrasted between the relevant conditions. In a second analysis, in order to obtain the time course of the activity on the source-level data, we identified two sources of interest in left temporal cortex corresponding to the peaks in activity difference between the related and unrelated conditions and between the related and identity conditions. The signals coming from these two sources were then averaged across the sources for each condition separately. This was done because an average of the two sources is a better characterisation of the left temporal cortex activity than from each one of the sources alone.

## Results

### Picture Naming Performance

The error rates were 2.2, 1.5, and .3% for the related, unrelated, and identity conditions, respectively. The log-odds of an incorrect response were 7.9 times higher in the related than in the identity condition (*β* = 2.01, *S.E.* = .38, *Wald Z* = 5.29, *p*<.001) and 1.5 times higher in the related than in the unrelated condition, although this effect was only marginally significant (*β* = .40, *S.E.* = .21, *Wald Z* = 1.94, *p* = .053). The mean naming RTs (95% confidence intervals (CI) around the mean in brackets), measured from picture onset, were 911 ms [904,918], 894 ms [887,901], and 831 ms [824,838] for the related, unrelated, and identity conditions, respectively. A main effect of distractor type was found by participants, *F*_1_(2,32) = 57.2, *p*<.001, and by items, *F*_2_(2,70) = 77.7, *p*<.001. Pictures paired with related distractors were named more slowly than pictures paired with unrelated distractors (Bonferroni corrected, by participants, *t*_1_(16) = 3.9, *p* = .002; by items, *t*_2_(35) = 2.5, *p* = .034, 95% *CI* [9], [30]) and more slowly than pictures paired with identity distractors (Bonferroni corrected, by participants, *t*_1_(16) = 9.7, *p*<.001; by items, *t*_2_(35) = 14.8, *p*<.001, 95% *CI* [64,100]). Furthermore, RTs were shorter in the identity than in the unrelated condition and participants became faster after the first stimulus presentation, but this decrease of RT was the same across conditions (see Figure S4).

### Induced Activity

#### Sensor level

The results of the sensor-level analyses are presented below for the stimulus- and response-locked activity.

##### Stimulus-locked activity

**Figure 1:**
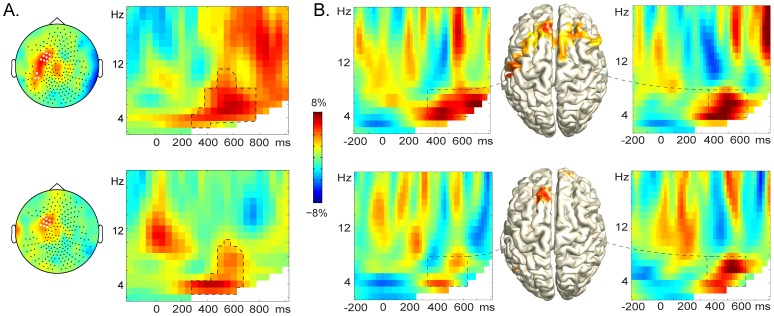
Induced brain responses. **A**. The panels in the right column show the stimulus-locked time-frequency representations of relative power change for Stroop-like (related vs. identity, upper right) and semantic (related vs. unrelated, lower right) effects, averaged over the sensors highlighted in white in the corresponding topographic maps to the left. Dashed lines indicate the clusters. **B**. The middle panel shows the estimated sources in the whole-brain analysis for the Stroop-like (upper) and semantic (lower) effects. The left and right panels show the time-frequency representation of the activity in the estimated sources. Dashed rectangles enclose the spectrotemporal cluster of interest (4–8 Hz, 350–650 ms). In this cluster, relative power increase was observed for the Stroop-like effect in the left superior frontal gyrus (upper right panel) and in the left postcentral gyrus (upper left panel). Relative power increase was observed for the semantic effect in the left superior frontal gyrus (lower right panel), but not in the left postcentral gyrus (lower left panel).

As presented in Figure 1A, the TFRs show relative power increase in the 4–10 Hz range between 350–650 ms in left-hemisphere sensors. For the stimulus-locked TFRs, using a cluster-based permutation approach that was frequency, time, and channel uninformed [45] while controlling for the false alarm rate, a statistically significant difference was revealed between the related and identity conditions (Stroop-like effect, upper TFR) that could be attributed to a spectro-spatio-temporal cluster of adjacent frequencies, time-points, and channels that exhibited similar power increases in the related relative to the identity condition (*p* = .028). Moreover, a statistically significant difference was revealed between the related and unrelated conditions (semantic effect, lower TFR) that could be attributed to a spectro-spatio-temporal cluster of adjacent frequencies, time-points, and channels that exhibited similar power increases in the related relative to the unrelated condition (*p* = .016). These clusters were detected roughly between 350–650 ms post-stimulus in the 4-10 Hz range over the sensors highlighted in white in the scalp topographies in Figure 1A (a more detailed characterisation of the clusters can be found in the Figure S1). Thus, the condition ordering of the theta power effect is in line with the ordering of mean RTs (related > unrelated; related > identity). Moreover, a negative correlation was observed between the induced activity and RTs in the related condition such that the higher the frontal theta-power was, the faster participants named the pictures (see Text S1). This result is in line with the hypothesis that the observed theta-power increase is related to resolving lexical competition. A theta-power increase was also observed for the unrelated relative to the identity condition (see Figure S2).

Analyses of the phase-locking factor [32] indicated that the power effects were not associated with differences in phase-locked responses to the stimulus (see Figure S5). Thus, this activity was likely induced by the stimulus as opposed to being evoked.

##### Response-locked activity

**Figure 2:**
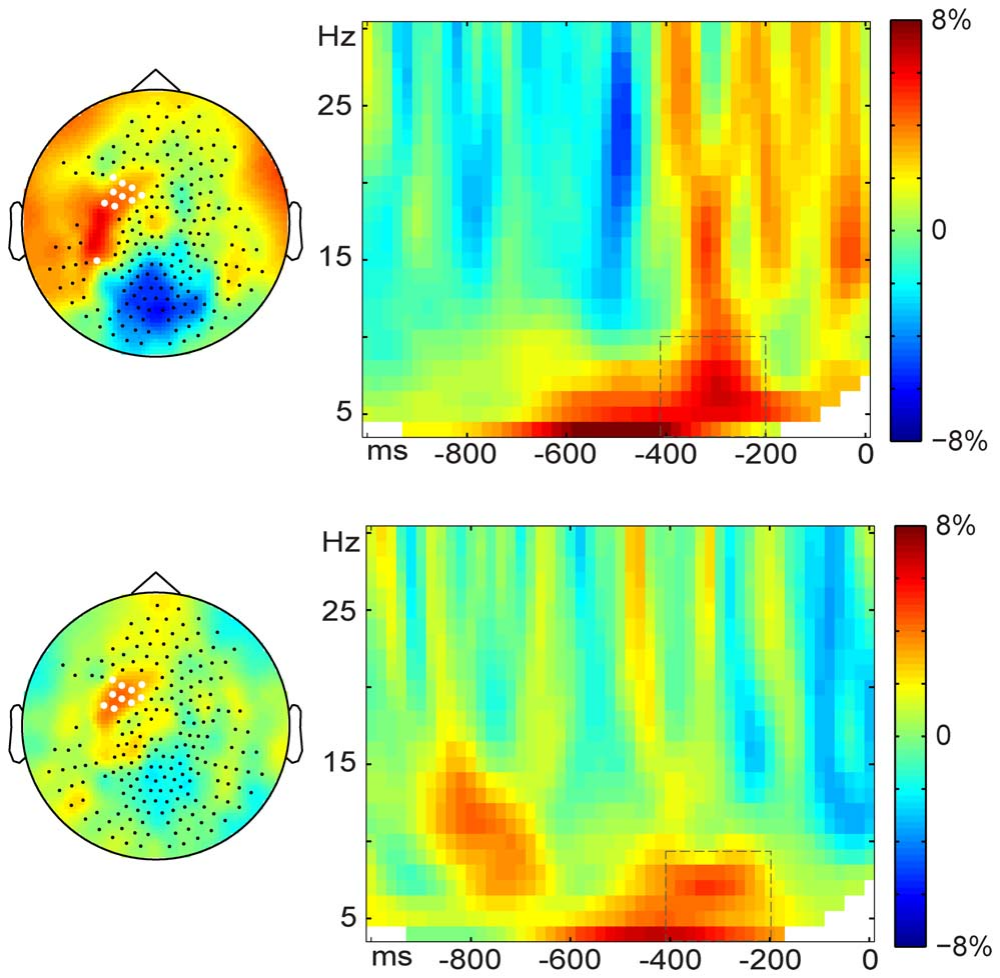
Induced brain responses time-locked to the onset of the naming responses. The right-hand panels show the response-locked time-frequency representations of relative power change for Stroop-like (related vs. identical, upper right) and semantic (related vs. unrelated, lower right) effects, averaged over the sensors highlighted in white in the corresponding topographic maps. Dashed lines indicate the clusters.

The response-locked analyses yielded a similar pattern of power changes as for the stimulus-locked activity. The TFRs presented in Figure 2 show relative power increase in the 4–10 Hz range between 400–200 ms before response onset. Significant spectro-spatio-temporal clusters were detected for the Stroop-like effect (*p* = .004) and for the semantic effect (*p* = .032). The condition ordering of the power effect is in line with the condition ordering of the mean RTs (related > unrelated; related > congruent). The convergence between stimulus- and response-locked analyses indicates that the TFR effects observed were not induced by differences in the onset of (preparation of) mouth movements between the conditions compared.

#### Source level

The estimated sources [51] of the Stroop-like effect, shown in the upper middle panel of Figure 1B, comprise the left postcentral gyrus [MNI peak activity: −50 −20 40] and the left superior frontal gyrus (SFG) [MNI peak activity: −10 30 50]. This latter source was also estimated for the semantic effect (lower middle panel of Figure 1B). The induced activity in these sources was estimated for each distractor-type effect [52]. In SFG, the averaged activity in the theta band (4–8 Hz) between 350–650 ms was significant for the Stroop-like effect (right upper panel of Figure 1B), *t*(13) = 2.4, *p* = .018, and for the semantic effect (right lower panel of Figure 1B), *t*(13) = 2.2, *p* = .025. In the postcentral gyrus, the averaged activity was significant for the Stroop-like effect (left upper panel of Figure 1B), *t*(13) = 2.1, *p* = .029, but non-significant for the semantic effect (left lower panel of Figure 1B), *p* = .216. Thus, the semantic and Stroop-like effects share a source in SFG. Importantly, the induced effects are significant already in the sensor-level analysis, but the source analysis corroborates the findings.

### Evoked activity

#### Sensor level

**Figure 3:**
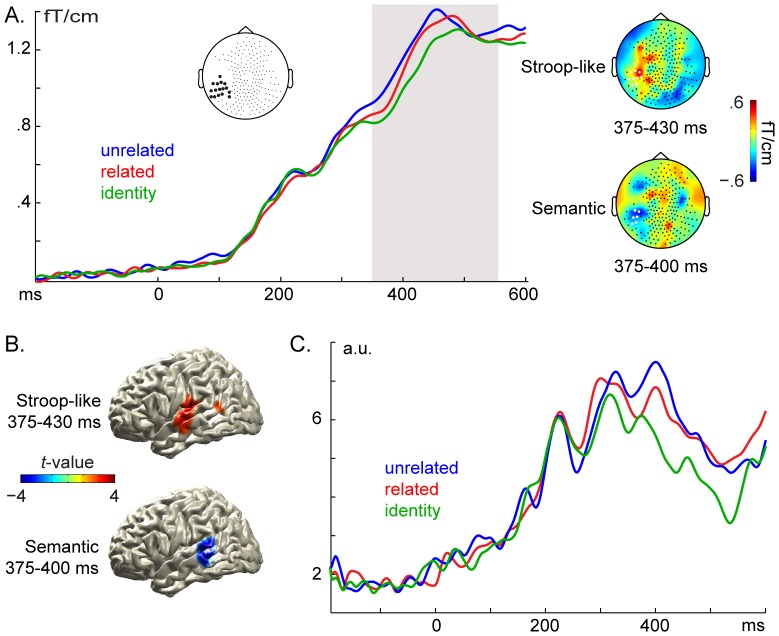
Evoked brain responses. **A**. Event-related fields (combined planar gradient) for the distractor types, averaged over the left temporal sensors highlighted in the layout in the middle. The grey area indicates the window tested for statistical significance. The Stroop-like effect (related vs. identity) was characterised by an amplitude increase in left temporal channels, as shown in the scalp topography to the right (upper topography), between 375–430 ms. The semantic effect (related vs. unrelated) was characterised by amplitude decrease in left temporal channels, as shown to the right (lower topography), between 375–400 ms. The scalp topographies show the difference between conditions averaged in the time window of the corresponding significant temporal cluster (shown below each topographical map) with the sensors participating in the cluster highlighted in white. **B**. Estimated sources of the Stroop-like (upper) and semantic (lower) effects in the whole-brain analysis in the time window of the corresponding significant temporal cluster (shown to the left of each source map). The difference *t* –value maps were thresholded at ±2.16 (13 degrees of freedom, alpha = .05). **C**. Activity from the left temporal cortex (averaged over the estimated sources in B) for the distractor types.

As expected, a peak around 450 ms after picture-word onset was observed in left-temporal sensors, as shown in Figure 3A. Using a time and sensor informed (350–550 ms, grey area in Figure 3A; left temporal sensors highlighted in black in the left layout) non-parametric cluster-based permutation test [45], we observed a statistically significant difference between the related and identity conditions that could be attributed to a spatio-temporal cluster of adjacent time-points and channels that exhibited a larger ERF amplitude for the related than for the identity conditions (*p* = .008). This cluster was detected between 375 ms and 430 ms over the sensors highlighted in white in the upper right topography. Moreover, a statistically significant difference was revealed between the related and unrelated conditions that could be attributed to a spatio-temporal cluster of adjacent time-points and channels that exhibited a smaller ERF amplitude for the related than for the unrelated conditions (*p* = .032). This cluster was detected between 375 ms and 400 ms over the sensors highlighted in white in the lower right topography. The topographical maps of the amplitude differences are shown to the right for the Stroop-like (upper map) and semantic (lower map) effects. Similar effects were observed when the onset of EMG activity from the mouth was used to determine the duration of the segments analysed. Finally, a smaller amplitude was obtained for the identity than for the unrelated condition (see Figure S3). These results indicate an N400m component, the ERF equivalent of the N400 [46], and are in line with the predicted relative effort of processing the picture-word stimuli.

#### Source level

Figure 3B presents the sources for the Stroop-like (upper) and semantic (lower) effects in the time windows identified in the sensor-level analyses (i.e., 375–430 ms for the Stroop-like effect and 375–400 ms for the semantic effect). As can be seen, the estimated sources comprise left superior and middle temporal cortex. Note that, due to the relatively limited spatial resolution of the source localisation of MEG data, the two sources in Figure 3B should not be interpreted as different sources for the Stroop-like and semantic effects. The signals from these two sources were then extracted and averaged over the two sources for each condition separately. As shown in Figure 3C, the distractors modulated the activity in these sources roughly between 300–500 ms after picture-word onset, with a peak around 400 ms. Note that the source analysis corroborates the sensor-level results but it does not imply that left temporal cortex is the only source of the N400m component in picture naming.

## Discussion

As outlined previously, a prominent theory of word production holds that word retrieval involves the activation of a set of candidate words in left middle temporal cortex, and a competitive selection of the intended word from this set regulated by frontal cortical mechanisms [1]–[5]. Previous electrophysiological studies reporting an N400 effect [8], [21], [25], examining only evoked brain activity, have provided evidence for the activation of multiple alternative words, but have not identified brain responses reflecting the competition caused by the activation of multiple alternatives. Furthermore, although previous fMRI studies have shown the involvement of frontal cortex in competition resolution, little is known about the time course of its involvement. The present results characterised a neuronal substrate associated with competition as well as its broad time course. Competition was reflected by induced activity, localised to left superior frontal gyrus (SFG), showing an oscillatory power increase in the 4–10 Hz range between 350–650 ms. Activity was larger for the related than unrelated and identity conditions, suggesting different degrees of effort in resolving the competition among the alternative words, as reflected in the RTs.

Additionally, we observed evoked brain activity in left temporal cortex (including MTG) showing differential modulation peaking around 400 ms after picture-word onset. Activity was larger for the unrelated than related and identity conditions, suggesting different degrees of effort (priming) in processing the candidate words activated by the picture-word stimuli. This latter finding is in line with both the competitive and noncompetitive accounts, which propose that in the related condition, picture and word prime each other (e.g. [2], [3], [9]). The observed sensor-level evoked brain activity agrees with previous ERP studies of picture-word interference [8], [21], [25], [26] and the prevailing processing-effort interpretation of the N400 effect [23], [24]. Moreover, in agreement with previous reports of the generators of the N400 in language comprehension [24], [53] and lexical activation in language production [1], [2], [31], [37], [38], the distractor-type modulations were observed in a brain area comprising the left MTG. The finding of attenuated activity for the related condition relative to the unrelated condition also agrees with fMRI findings showing reduced left MTG activity for related relative to unrelated picture-word stimuli [54]. Although this activity could also be related to the activation of concepts, the left MTG source is more compatible with lexical activation rather than the activation of concepts [37], [55]. Our results show that the evoked and induced brain activity largely overlap in time, although they are differentially modulated by the distractor words and associated with different brain sources.

The observed induced activity in the theta band, localized to the left SFG (possibly also including the most anterior portion of the supplementary motor area (pre-SMA) and the anterior cingulate cortex (ACC)), agrees with previous findings on executive control processes in various frontal areas [41], [56]–[60]. Theta oscillations have moreover been associated with manipulations of task-relevant information by executive control processes [40], [57]–[59]. For example, theta-band effects in the ACC have previously been observed in manual Stroop task performance, where power increased with increasing competition between 400 and 800 ms after stimulus onset [40]. Theta-band effects have also been observed in a word production study employing a semantic blocking task ([39], but see ref. [26] for a report of beta-band effects). Although the spatial resolution of our source analyses using MEG is relatively low compared to fMRI [52], [61], [62], our frontal source also agrees with previous fMRI studies, which related activity in the left SFG and pre-SMA to effort in lexical selection [63], and activity in the left SFG to competition in Stroop-like tasks [41], [64]. Moreover, lesion-deficit analyses have related bilateral SFG to impaired performance on the colour-word Stroop task [56] and the left SFG to executive control processes in working memory [60].

Moreover, our findings seem to agree with EEG evidence from Ewald et al. [39] using a semantic blocking paradigm, who demonstrated that semantic interference effects in word production are associated with functional connectivity in the theta frequency band (7 Hz) between frontal and posterior areas. It should be noted, however, that the functional connectivity in the theta frequency band reported by Ewald et al. is a physiological phenomenon that is likely different from the theta-power modulations we report in our study. Their analysis reflects functional connectivity, by definition a phenomenon involving distant brain regions, while our findings reflect a local phenomenon (i.e., within a brain region). An additional clear difference with our study is that we report power-modulations to occur prior to articulation onset. The mere overlap in frequency range does not suffice for making these two phenomena similar to each other. Furthermore, the posterior areas of Ewald et al. concerned occipito-temporal areas, whereas we observed effects in more anterior temporal areas. Our findings also seem to agree with EEG evidence from Aristei et al. [29], who reported semantic interference ERP effects (related > unrelated) at left temporal channels (semantic blocking effects) and left frontal channels (distractor word effects), in line with previous fMRI studies [35]. However, in the study of Aristei et al., no source reconstruction was performed so all the reported effects are on the scalp level. Given the problems with volume conductance known for EEG [65], no inferences can be made regarding brain regions for the study of Aristei et al. Moreover, whereas Aristei et al. found ERP modulations in frontal channels, our frontal modulation was associated with the induced activity only. Therefore, it is somewhat hard to make direct comparisons between their effects and what we obtained. Nevertheless, our present findings seem to broadly agree with the previous EEG findings of Ewald et al. and Aristei et al.

The resolution of lexical competition has also been associated with the left inferior frontal gyrus (LIFG) in both fMRI and lesion-deficit analyses [35] using the blocked-cyclic naming paradigm, which was not found to be active in the present study. It should be noted that activity in the LIFG has been found in some fMRI studies of picture-word interference ([66], see also ref. [67] for a modified version of this task], but certainly not all [54], [68], [69]). It is possible that the present MEG study was insufficiently powerful or sensitive to detect the activity in the LIFG. Alternatively, it may be that the picture-word interference task engages the LIFG less strongly than the blocked-cyclic naming task, perhaps because it does not rely on the same top-down mechanisms for selection as blocked-cyclic naming does [70], an issue that may be examined in future studies. Crucially, previous fMRI and lesion-deficit analyses [35], [54], [66], [67] did not identify the temporal relation between left MTG activity (lexical activation processes) and frontal activity (competition resolution processes). The present results generally agree with existing findings, but importantly, provide evidence on the temporal dynamics of left temporal and left frontal activity, suggesting a tight temporal link between the two. The tight temporal relation between these two activities is in line with an account in terms of lexical activation and competition resolution [1]–[5].

The modulations of brain activity reported here (around 400 ms in the evoked activity) appear rather late in comparison to some previous findings on evoked activity associated with language production [21], [29], [30], [31] although they are in line with other findings [8], [21], [25], [71]. Note that the early evoked responses reported by Dell'Acqua et al. [21] were associated with early visual processing of the distractor word, whereas activity in the N400 time window was interpreted in terms of lexical activation [21], in line with our interpretation and the interpretation of Blackford et al. [8]. Moreover, Aristei et al. [29] did not have *visual* but spoken distractors and Costa et al. [30] and Maess et al. [31] did not employ the picture-word interference paradigm. Timing estimates of lexical selection [37], [38], [72] are based on studies of picture naming without visual word distractors. Picture-naming RTs in the picture-word interference task are typically 100 to 200 ms longer than in standard picture naming (e.g. [16], [21], [26], [73]). Thus, it is plausible to assume that the presence of visual distractors in picture-word interference prolongs the visual perceptual processing of the picture, thereby also delaying the onset of lexical selection [26], [38]. Under this assumption, the timing of the reported modulations is in line with previous studies. The prolonging of perceptual processing of the picture may be due to visual load as well as due to the effort required for object identification. It seems plausible to assume that visual processing of the picture is hampered more by visual than spoken word distractors.

### Evaluating the Noncompetitive Account

We associated the evoked and induced brain activity with, respectively, the activation of a set of candidate words and the competitive selection of the intended word from this set. The tight temporal link between these two activities, and their timing relative to articulation onset, is especially important in light of an alternative account of word retrieval [9], [10], [27], according to which a word is selected if its activation exceeds some threshold, but selection is assumed to be independent of the activation state of other words. The semantic effect is assumed to arise after word planning, reflecting the exclusion of a motor programme for the distractor word from an articulatory buffer [9], [27]. This exclusion process is assumed to take longer when the distractor is semantically related to the picture than when it is unrelated, yielding the semantic interference effect in the naming RTs.

Previous fMRI studies [54], [66] could not adjudicate between the competitive and noncompetitive accounts because no precise time information is obtained with this method. However, our results of the response-locked analyses do help adjudicate between the two accounts. According to the noncompetitive response-exclusion account, the interference effect emerges at the point of deciding between the motor programmes of the target and distractor in the output buffer [10], [27]. Thus, interference arises when the motor programme has been derived for the picture and the programme for the distractor word is in the buffer. The presumed greater difficulty of deciding between motor programmes in the related than unrelated condition yields the semantic interference in RTs. Note that this decision process could be regarded as an attentional mechanism associated with a source in prefrontal cortex. However, according to time estimates from meta-analyses [37], [38], picture name planning reaches the articulatory buffer no earlier than about 145 ms before articulation onset. Thus, according to the noncompetitive account, brain activity that reflects interference (i.e., activity that is in line with the condition ordering of RTs) should not occur earlier than about 145 ms before speech onset. However, the modulations of oscillatory power observed in our response-locked analyses already occurred between 400 and 200 ms before articulation onset, which is too early to be in agreement with the noncompetitive account.

According to a different version of the response-exclusion account, the removal process starts as soon as the motor programme for the distractor reaches the articulatory buffer: “When the response to the distractor still occupies the buffer when the response to the picture becomes available, picture naming has to be postponed until the initial response is purged from the buffer” (ref. [28], p. 887). One could perhaps argue that the induced brain activity that we observed reflects this immediate removal process rather than reflecting the decision between two motor programs in the buffer only. Dhooge and Hartsuiker [28] observed that when a distractor word is presented 200 ms before picture onset, the distractor word still affects picture naming RTs (with mean picture naming RTs around 600 ms). This effect can only be obtained in the RTs if the exclusion process is still ongoing when picture name planning reaches the buffer, which is around 455 ms after picture onset (with a mean RT of 600 ms, ref. 37). This implies that the exclusion process takes at least some 655 ms (i.e., 455+200 ms) from the moment that the motor programme for the distractor reaches the buffer. This prediction is also not borne out by our data, which indicate that the induced activity is confined to a restricted time window, between 350 and 650 ms after picture onset. It could be argued that the interference effects in the RTs arise not only due to competition but also due to other psychological phenomena, which at this point are still undefined. Thus, as long as other hypotheses are not formulated, lexical competition and response exclusion remain the two testable hypotheses, with the present findings supporting the competition hypothesis.

To conclude, our findings are not in agreement with any of the versions of the response exclusion account in the literature (i.e. [9], [10], [27], [28]). This is in line with the accumulating empirical evidence against this hypothesis (e.g. [19], [20], [73]–[85]).

### Evaluating the Competition Account by Computer Simulations

Blackford et al. [8] stated that “the electrophysiological evidence for semantic priming in the presence of behavioral interference provides evidence against an account of selection by competition at the lemma level” (p. 97). They assumed that the picture name is primed by the distractor word. However, we assume that, in addition, the distractor word is primed by the picture (i.e., reverse priming, making related words more potent competitors than unrelated words). This is in line with the evidence that both pictures and words evoke an N400 response [23], [24]. Using the WEAVER++ model of word production, Roelofs [16] presented the results of computer simulations demonstrating that the semantic interference effect in RTs can be explained by reverse priming combined with the assumption that a word becomes available for selection only if its activation exceeds that of competitor words by a critical amount (the response threshold). Moreover, computer simulations by Roelofs et al. [86] using this model demonstrated that if frontal cortex is involved in top-down enhancing the activation of the target until its activation exceeds the selection threshold, the patterns of frontal activity typically observed in Stroop-like tasks are explained.

**Figure 4:**
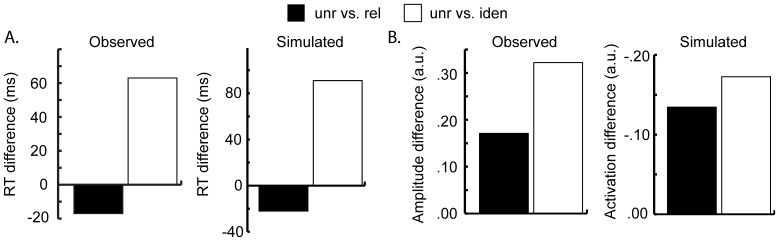
Observed results and WEAVER++ simulations. **A**. Differences in picture-naming times as empirically observed and from the simulations for the related condition (black bar) and identity condition (white bar) relative to the unrelated condition. **B**. Differences in signal amplitude of left temporal cortex activity for the related condition (black bar) and identity condition (white bar) relative to the unrelated condition and corresponding priming effects in the simulations. RT =  response time; unr =  unrelated; rel =  related; iden =  identity.

To demonstrate that this competitive-selection account explains the electrophysiological evidence for semantic priming in the presence of behavioural interference in the present study, we conducted computer simulations using WEAVER++. The simulation protocol and parameters were exactly the same as in earlier simulations using the model (e.g. [2]–[4], [16], [86]) except that the response threshold was set at 2.0 to fine-tune the fit to the data. The results of the simulations along with the present empirical results are shown in Figure 4. In line with the observed results, the model yields longer RTs for the related than for the unrelated condition and shorter RTs for the identity than for the unrelated condition (Figure 4A). Moreover, in line with the observed results, the model yields more priming in the identity than in the related condition, and both conditions show more priming than the unrelated condition (Figure 4B). Priming in the model is depicted as the difference in peak activation between conditions. The simulation results corroborate our account of the present findings in terms of lexical activation and competition.

To conclude, we obtained evidence that evoked (i.e., phase-locked) activity in left temporal cortex and induced (i.e., non-phase-locked) activity in superior frontal cortex, respectively, characterise lexical activation and competitive selection in overt picture naming. These findings support the theory of lexical selection by competition.

## Supporting Information

Figure S1 **Temporal and spectral extension of the significant cluster of the induced activity for the Stroop-like (panel A) and semantic (panel B) effects.**
(TIF)

Figure S2 **Induced brain responses time-locked to the onset of the stimulus.** The right-hand panel shows the time-frequency representation of relative power change for the contrast unrelated vs. identity averaged over the significant sensors (as reported in the main article). To the left, the scalp topography of the significant theta cluster is shown.
(TIF)

Figure S3 **Scalp topography of the contrast unrelated vs. identity, averaged over the time window of the corresponding significant temporal cluster (350–423 ms).**
(TIF)

Figure S4 **Mean naming response times (RTs) as a function of distractor type and repetition.** Error bars indicate 95% confidence intervals around the mean, calculated from the variance over participants.
(TIF)

Figure S5 **Phase-locking factor.** The panels in the right column show the stimulus-locked PLF for Stroop-like (related vs. identity, upper right) and semantic (related vs. unrelated, lower right) effects, averaged over the sensors highlighted in the topographic maps to the left. RT =  response times; iden  =  identity condition; re =  related condition; unr =  unrelated condition.
(TIF)

Table S1 **Stimulus list.** English translations in parentheses.
(DOCX)

Text S1 (DOC)
