# Supplementary figures and images for: Distinct Patterns of Brain Activity Characterise Lexical Activation and Competition in Spoken Word Production
Source: PLoS One. 2014 Feb 18;9(2):e88674. doi: 10.1371/journal.pone.0088674 (PMC3928283; doi:10.1371/journal.pone.0088674)

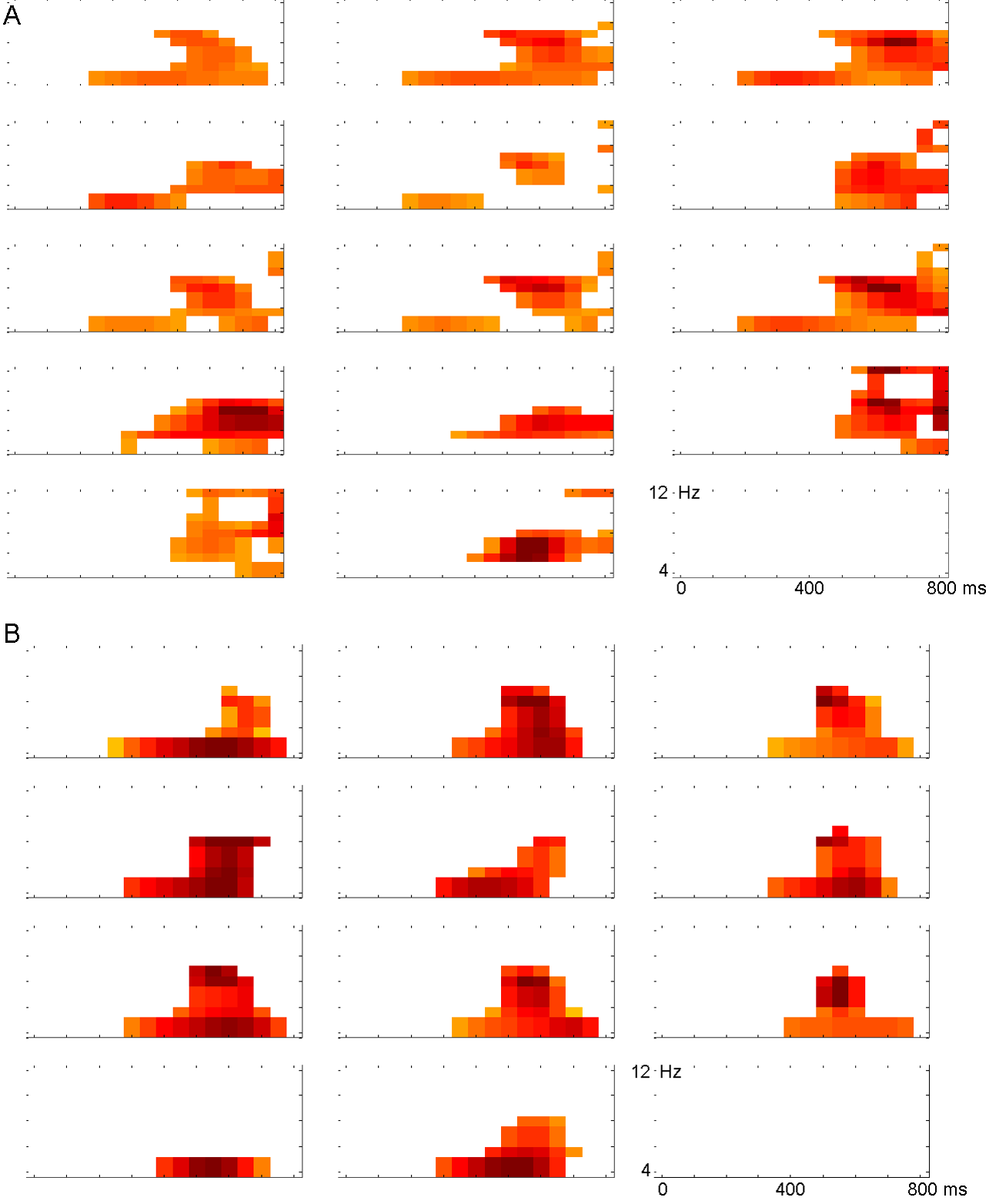

Supplement: Figure S1 — Temporal and spectral extension of the significant cluster of the induced activity for the Stroop-like (panel A) and semantic (panel B) effects. (TIF) [file pone.0088674.s001.tif]

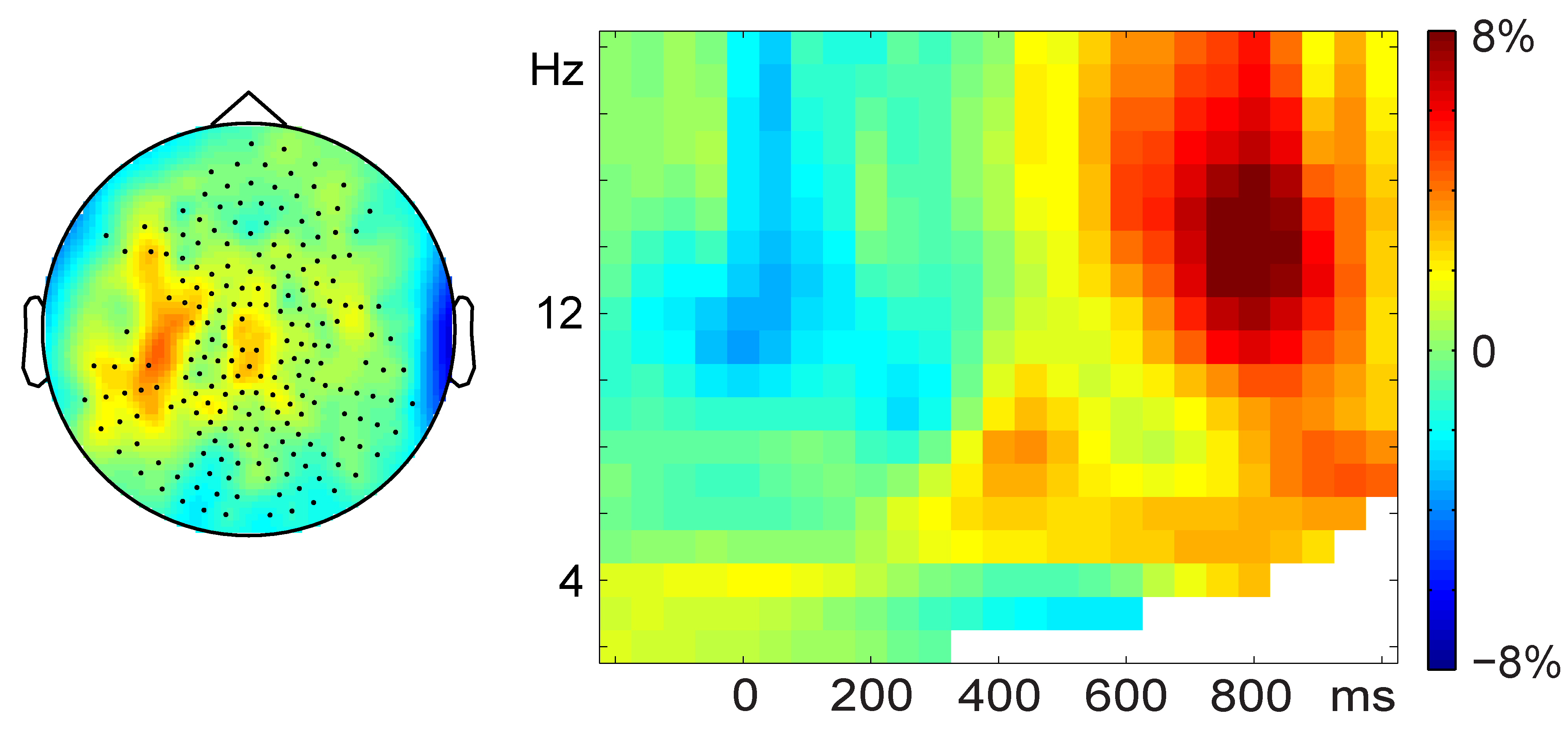

Supplement: Figure S2 — Induced brain responses time-locked to the onset of the stimulus. The right-hand panel shows the time-frequency representation of relative power change for the contrast unrelated vs. identity averaged over the significant sensors (as reported in the main article). To the left, the scalp topography of the significant theta cluster is shown. (TIF) [file pone.0088674.s002.tif]

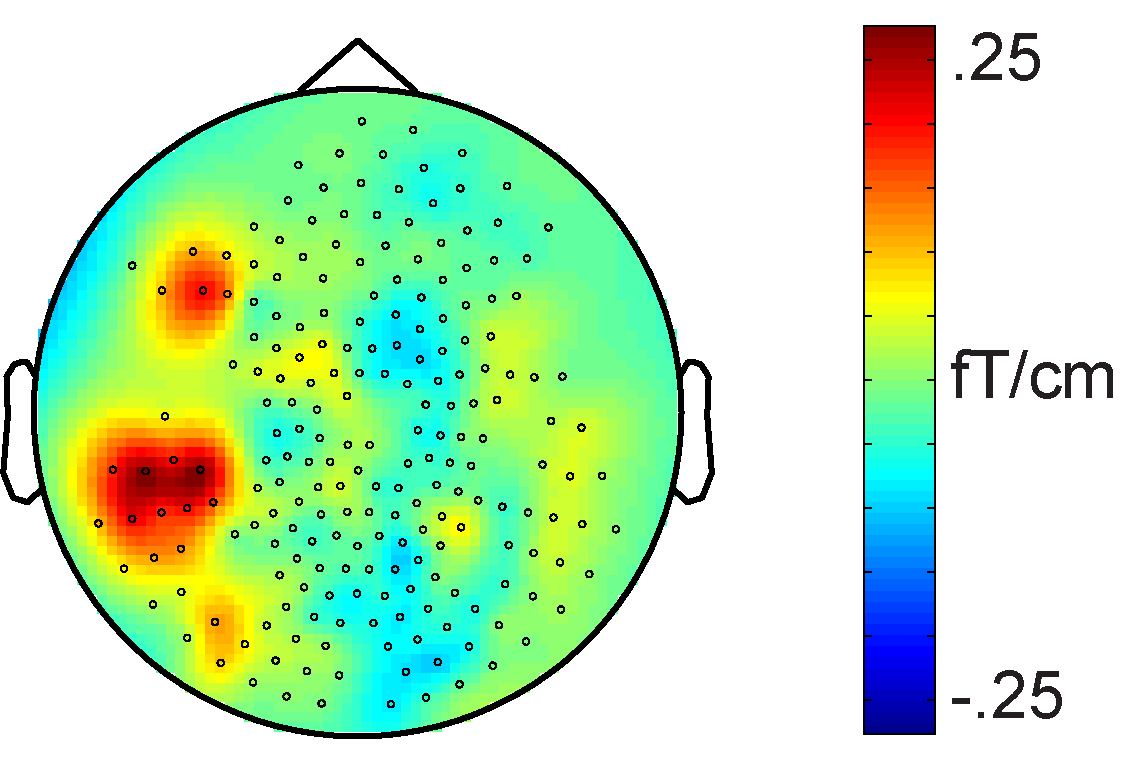

Supplement: Figure S3 — Scalp topography of the contrast unrelated vs. identity, averaged over the time window of the corresponding significant temporal cluster (350–423 ms). (TIF) [file pone.0088674.s003.tif]

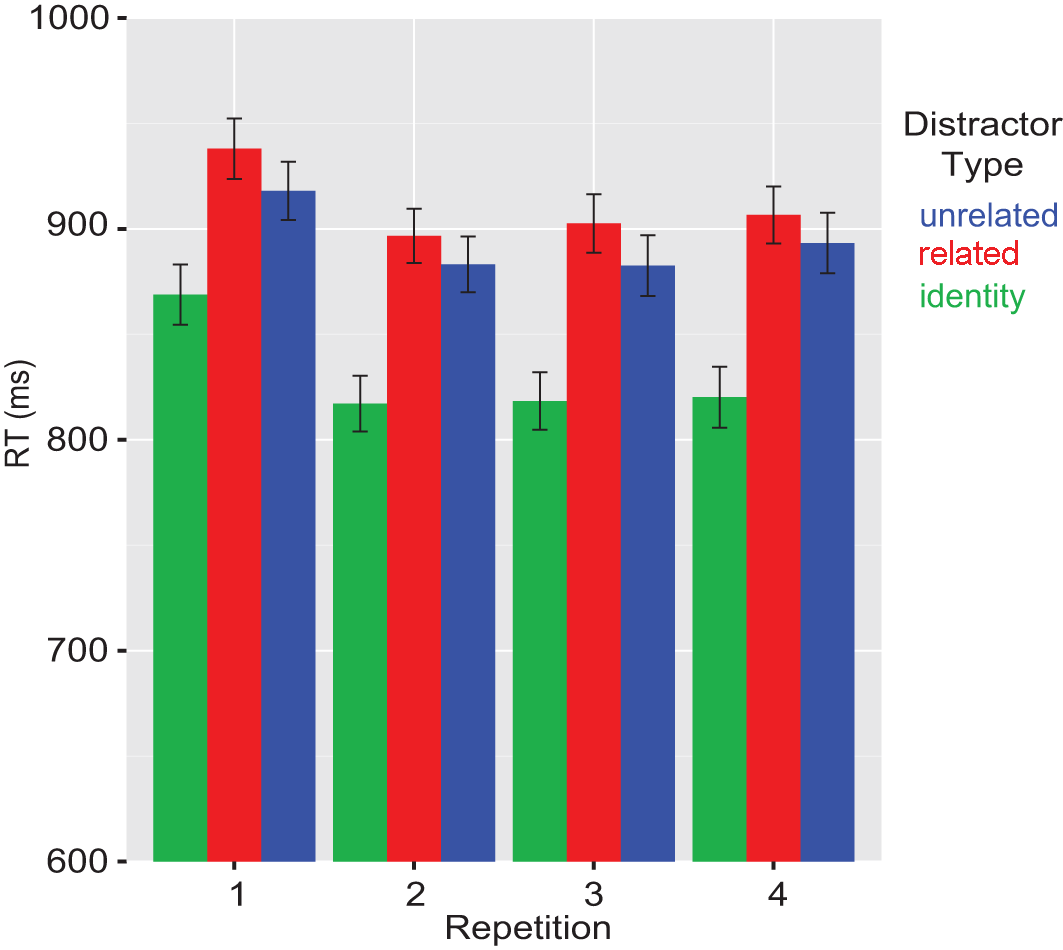

Supplement: Figure S4 — Mean naming response times (RTs) as a function of distractor type and repetition. Error bars indicate 95% confidence intervals around the mean, calculated from the variance over participants. (TIF) [file pone.0088674.s004.tif]

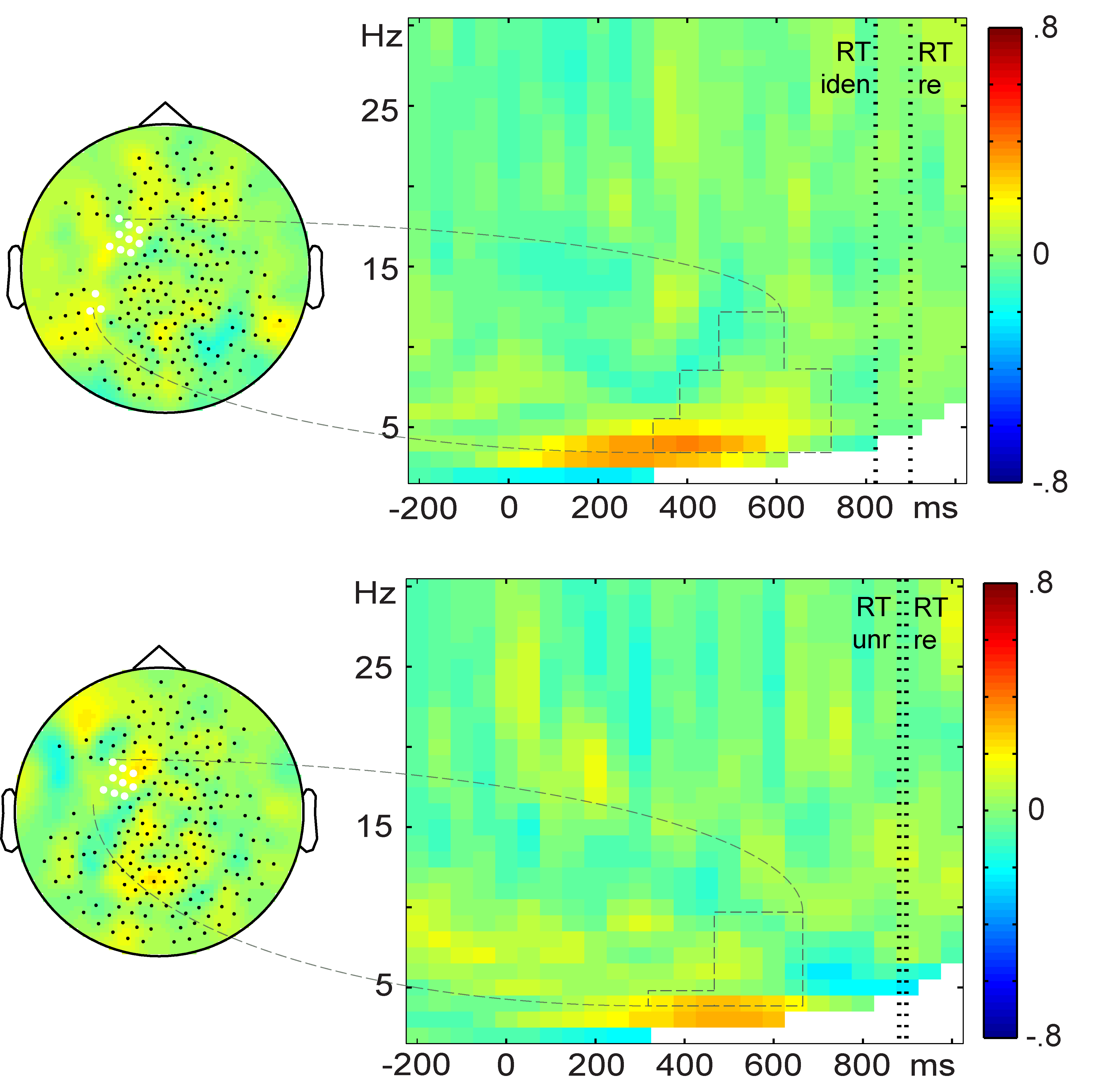

Supplement: Figure S5 — Phase-locking factor. The panels in the right column show the stimulus-locked PLF for Stroop-like (related vs. identity, upper right) and semantic (related vs. unrelated, lower right) effects, averaged over the sensors highlighted in the topographic maps to the left. RT = response times; iden = identity condition; re = related condition; unr = unrelated condition. (TIF) [file pone.0088674.s005.tif]
