# Supplementary material for: Distinct Patterns of Brain Activity Characterise Lexical Activation and Competition in Spoken Word Production
Source: PLoS One. 2014 Feb 18;9(2):e88674. doi: 10.1371/journal.pone.0088674 (PMC3928283; doi:10.1371/journal.pone.0088674)
Supplement: Table S1 — Stimulus list. English translations in parentheses. (DOCX) [file pone.0088674.s006.docx]

| Picture name | Related distractor | Unrelated distractor |
| --- | --- | --- |
| ananas (pineapple) | banaan | kasteel |
| appel (apple) | peer | dolk |
| arm (arm) | neus | trein |
| auto (car) | bus | konijn |
| banaan (banana) | ananas | trui |
| bed (bed) | tafel | pistool |
| been (leg) | oor | auto |
| beker (cup) | kan | geit |
| bord (plate) | glas | kanon |
| bureau (desk) | kast | hert |
| bus (bus) | auto | glas |
| dolk (dagger) | zwaard | appel |
| fabriek (factory) | kerk | neus |
| fiets (bicycle) | trein | kast |
| geit (goat) | zwaan | beker |
| glas (glass) | bord | bus |
| hemd (shirt) | jas | oor |
| hert (deer) | konijn | bureau |
| jas (jacket) | hemd | peer |
| kan (jug) | beker | molen |
| kanon (cannon) | pistool | bord |
| kast (wardrobe) | bureau | fiets |
| kasteel (castle) | molen | ananas |
| kerk (church) | fabriek | been |
| konijn (rabbit) | hert | arm |
| molen (mill) | kasteel | kan |
| neus (nose) | arm | fabriek |
| oor (ear) | been | hemd |
| peer (pear) | appel | jas |
| pistool (pistol) | kanon | bed |
| rok (skirt) | trui | zwaan |
| tafel (table) | bed | zwaard |
| trein (train) | fiets | kerk |
| trui (sweater) | rok | banaan |
| zwaan (swan) | geit | rok |
| zwaard (sword) | dolk | tafel |
