# Supplementary material for: Distinct Patterns of Brain Activity Characterise Lexical Activation and Competition in Spoken Word Production
Source: PLoS One. 2014 Feb 18;9(2):e88674. doi: 10.1371/journal.pone.0088674 (PMC3928283; doi:10.1371/journal.pone.0088674)
Supplement: Text S1 — (DOC) [file pone.0088674.s007.doc]

**Supplement**

**Materials**

The stimulus list is shown in Supplementary Table S1. The pictures were selected from the picture database of the Max Planck Institute for Psycholinguistics, Nijmegen, together with their basic-level names in Dutch. Experiments with comparable sets of materials from the same database have been previously reported in Piai et al. (2011, Experiment 1), Piai et al. (2012b, Experiment 2), Piai and Roelofs (2013), Roelofs (2006, Experiment 1B), and Roelofs (2007, Experiment 1).

**Significant clusters in the statistical analysis of the induced activity**

Figure S1 shows the temporal and spectral extension of the cluster for the Stroop-like (panel A) and semantic (panel B) effects. Note that the temporal extent of the clusters at some sensors exceeds the time window we report in the main text as significant (i.e., 350-650 ms). We refer the reader to Maris (2012) for a detailed discussion regarding inferences about the temporal (and spectral) dimension of the cluster.

**Unrelated and Identity Contrast**

For completeness, below we report the behavioural and sensor-level results for the comparison between unrelated and identity distractor types.

**RTs.** Pictures paired with unrelated distractors were named more slowly than pictures paired with identity distractors (by participants*, t*_1_(16) = 6.6, *p* < .001; by items, *t*_2_(35) = 13.1, *p* < .001).

**Induced activity.** Relative power changes for the unrelated relative to the identity condition are shown in Supplementary Figure S2. A significant cluster was observed roughly between 400-600 ms in the 6-8 Hz range, *p* = .024. The scalp topography was derived from the relative power changes averaged over the spatio-spectro-temporal cluster.

**Evoked activity.** Supplementary Figure S3 shows the scalp topography of the differential activity between the unrelated and identity conditions. A temporal cluster was detected between 350 and 423 ms, *p* = .012, which is the time window from which the scalp topography was derived.

**Repetition Effect in the RTs**

In our study, we presented the same picture-word pairs four times each, which could be problematic given demonstrations that the N400 effect can be sensitive to repetition priming (e.g., Rugg 1985). However, there are reasons why the repetition of items in our study may not be a confounding factor. Firstly and importantly, our results replicate previous findings regarding the N400 modulations as a function of distractor type (i.e., unrelated > related > identity, Aristei et al. 2011; Blackford et al. 2012; Greenham et al. 2000; Hirschfeld et al. 2008; Piai et al. 2012a), suggesting that the repetition of the items in our study did not affect the N400 effect differentially as a function of distractor type. Secondly, previous studies examining the effect of repetition in picture naming have reported that the repetition of items affected the ERPs in a similar manner across conditions (Aristei et al. 2011; Strijkers et al. 2010).

Finally, we submitted the naming RTs to analyses of variance on the average naming RTs across participants (*F*_1_) and across items (*F*_2_), with distractor type and repetition as independent variables to evaluate the behavioural effects of picture-word repetition. The RTs as a function of distractor type and repetition are shown in Supplementary Figure S4. A main effect of repetition was found by participants, *F*_1_(3,48) = 20.9, *p* < .001, and by items, *F*_2_(3,105) = 39.7, *p* < .001. Importantly, the change in naming latencies as a function of repetition was equal across distractor types, *F*_1_(6,96) = 1.2, *p* = .289; *F*_2_(6,210) = 1.0, *p* = .398.

In sum, altogether, there seems to be no indication that the repetition of the picture-word pairs is a confounding factor in our study.

**Correlational Analysis between RTs and Induced Activity**

To better assess the relation between behaviour and brain activity, we correlated the induced left-frontal theta activity with the RTs for the related condition, which is the condition in which the enhanced competition prolongs word-selection duration relative to other conditions, thus yielding the semantic and Stroop-like interference effects in the RTs. If the observed increase in theta power relates to resolving lexical competition, we would expect that the higher the power on a given trial, the shorter the naming RT (i.e., a negative correlation). On the group level, if in general there is a negative correlation between averaged power and RT across participants, then the mean of the correlation coefficients over participants should be significantly smaller than zero.

For this analysis, the mean power within the frontal-theta cluster was calculated on a single trial level (cluster: 350-650 ms, 4-7 Hz, significant channels shown in Figure 1A (main article). The upper margin of 7 Hz was chosen because the power modulations were the strongest within the theta band, i.e., 4-7 Hz). For each participant, the Spearman's rank correlation coefficient was calculated between the power and the RT across trials. To assess the relation between RT and induced activity on the group level, the participants’ coefficients were tested with a one-sample *t*-test (one-tailed) against zero (see for a similar approach Cohen and Cavanagh, 2011).

For the theta cluster between 350-650 ms, the correlation coefficients significantly differed from zero in the related condition, *rho* = -.08, *t*(16) = -2.06, *p* = .03. (The *rho* coefficient here is the averaged coefficient value over participants.)

To show that this correlation is specific to the observed left-frontal cluster in the theta band, we conducted a control analysis similar to the above, but with the power calculated over the right hemisphere counterparts of the significant cluster of sensors (time window and frequency band were kept the same). In this analysis, there was no correlation between theta-frontal power and RTs, *rho =* -.02, *t*(16) = -.58, *p* = .28. Thus, the higher the left-frontal theta power, the shorter the RT (in the related condition). These results corroborate the evidence from the distractor type effects (see main article) that the observed increase in left-frontal theta power between 350 and 650 ms is associated with resolving lexical competition.

**Phase-Locking Analysis**

To ensure that the power effects were not associated with differences in phase-locked responses to the stimulus, phase adjustment prior to the onset of stimulus presentation was assessed for each participant using the phase-locking factor (PLF, Tallon-Baudry et al. 1996). The PLF over N trials is defined as:

where *φ^k^( f_0_, t)* corresponds to the estimated phase at frequency *f_0_* and time *t* resulting from the time-frequency analysis.

A PLF close to 0 reflects strong phase variability, whereas a PLF of 1 reflects that all trials exhibit the same phase at a given frequency and point in time. As for the TFR analysis of power, we calculated the PLF with respect to a sliding time window of four cycles between 2 and 30 Hz, advanced in steps of 50 ms and of 1 Hz. A Hanning taper was then applied to each time window before estimating phase with the Fast Fourier transform. We computed the PLF for both the horizontal and vertical components of the estimated planar gradients and combined the two measures by taking their average.

**Statistical Analysis of the Phase-Locking Factor**

We tested the significance of the differences in PLF between conditions. To this end, the activity of interest (time x frequency x channel) was determined based on the significant clusters detected in the statistical analysis of the stimulus-locked TFRs (4-10 Hz and 350-650 ms). Next, a cluster-based permutation test (Maris and Oostenveld 2007) was performed on the activity of interest to determine whether the TFR effects obtained were phase-locked or not.

Supplementary Figure S5 shows the stimulus-locked PLF for Stroop-like (upper, related vs. identity) and semantic (lower, related vs. unrelated) effects, averaged over the sensors that were significant in the TFRs (highlighted in the topographic maps to the left). The difference in PLF was calculated by subtracting the PLF of the related condition from the PLF of the identity condition (Stroop-like effect) or unrelated condition (semantic effect). The figure suggests no consistent differences in PLF between conditions in the frequency of interest. Supplementary Figure S5 also shows the topographic maps for the PLF associated with Stroop-like (upper) and semantic (lower) effects, averaged over the time and frequency ranges of interest (enclosed in dashed lines). The scalp topographies suggest no differences in PLF between the conditions compared. No significant clusters were detected in the PLF between conditions, Stroop-like effect (related vs. identity), *p* = .323, semantic effect (related vs. unrelated), *p* = .627.

**References**

Aristei S, Melinger A, Abdel Rahman R. 2011. Electrophysiological chronometry of

semantic context effects in language production. J Cogn Neurosci. 23:1567–1586.

Blackford T, Holcomb P J, Grainger J, Kuperberg G R. 2012. A funny thing happened on

the way to articulation: N400 attenuation despite behavioral interference in picture naming. Cognition. 123:84-99.

Cohen M X, Cavanagh J F. 2011. Single-trial regression elucidates the role of prefrontal

theta oscillations in response conflict. Front Psychology. 2:30.

Greenham S L, Stelmack R M, Campbell K B. 2000. Effects of attention and semantic

relation on event-related potentials in a picture-word naming task. Biol Psychol. 50:79-104.

Hirschfeld G, Jansma B, Bölte J, Zwitserlood P. 2008. Interference and facilitation in

overt speech production investigated with ERPs. Neuroreport. 19:1227-1230.

Maris E. 2012. Statistical testing in electrophysiological studies. Psychophysiol. 49:549-

565.

Maris E, Oostenveld R. 2007. Nonparametric statistical testing of EEG- and MEG-data. J

Neurosci Meth. 164:177-190.

Piai V, Roelofs A. 2013. Working memory capacity and dual-task interference in picture

naming. Acta Psychol. 142:332-342.

Piai V, Roelofs A, Schriefers H. 2011. Semantic interference in immediate and delayed

naming and reading: Attention and task decisions. J Mem Lang. 64:404-423.

Piai V, Roelofs A, van der Meij R. 2012a. Event-related potentials and oscillatory brain

responses associated with semantic and Stroop-like interference effects in overt naming. Brain Res. 1450:87-101.

Piai V, Roelofs A, Schriefers H. 2012b. Distractor strength and selective attention in

picture naming performance. Mem Cognition. 40:614-627.

Roelofs A. 2006. Context effects of pictures and words in naming objects, reading words,

and generating simple phrases. Q J Exp Psychol. 59:1764-1784.

Roelofs A. 2007. Attention and gaze control in picture naming, word reading, and word

categorizing. J Mem Lang. 57:232-251.

Rugg M D. 1985. The effects of semantic priming and word repetition on event-related

potentials. Psychophysiology. 22:642-647.

Strijkers K, Costa A, Thierry G. 2010. Tracking lexical access in speech production:

electrophysiological correlates of word frequency and cognate effects. Cereb Cortex. 20:912-928.

Tallon-Baudry C, Bertrand O, Delpuech C, Pernier J. 1996. Stimulus specificity of phase-

locked and non-phase-locked 40 Hz visual responses in human. J Neurosci. 16:4240-4249.
